# Interleukin-4 Inhibits Regulatory T Cell Differentiation through Regulating CD103+ Dendritic Cells

**DOI:** 10.3389/fimmu.2017.00214

**Published:** 2017-03-03

**Authors:** Lei Tu, Jie Chen, Hongwei Zhang, Lihua Duan

**Affiliations:** ^1^Division of Gastroenterology, Union Hospital, Tongji Medical College, Huazhong University of Science and Technology, Wuhan, China; ^2^Medical College, Xiamen University, Xiamen, China; ^3^Laboratory of Clinical Immunology, Wuhan No. 1 Hospital, Tongji Medical College, Huazhong University of Science and Technology, Wuhan, China; ^4^Department of Rheumatology and Clinical Immunology, The First Affiliated Hospital of Xiamen University, Xiamen, China

**Keywords:** IL-4, dendritic cells, CD103, Treg, indoleamine 2,3-dioxygenase, inflammatory bowel diseases

## Abstract

CD103+ dendritic cells (DCs) have been shown to play a crucial role in the pathogenesis of inflammatory bowel diseases (IBDs) through educating regulatory T (Treg) cells differentiation. However, the mechanism of CD103+ DCs subsets differentiation remains elusive. Interleukin (IL)-4 is a pleiotropic cytokine that is upregulated in certain types of inflammation, including IBDs and especially ulcerative colitis. However, the precise role of IL-4 in the differentiation of CD103+ DCs subpopulation remains unknown. In this study, we observed a repressive role of IL-4 on the CD103+ DCs differentiation in both mouse and human. High-dose IL-4 inhibited the CD103+ DC differentiation. In comparison to CD103− DCs, CD103+ DCs expressed high levels of the co-stimulatory molecules and indoleamine 2,3-dioxygenase (IDO). Interestingly, IL-4 diminished IDO expression on DCs in a dose-dependent manner. Besides, high-dose IL-4-induced bone marrow-derived DCs, and monocyte-derived DCs revealed mature DCs profiles, characterized by increased co-stimulatory molecules and decreased pinocytotic function. Furthermore, DCs generated under low concentrations of IL-4 favored Treg cells differentiation, which depend on IDO produced by CD103+ DCs. Consistently, IL-4 also reduced the frequency of CD103+ DC *in vivo*. Thus, we here demonstrated that the cytokine IL-4 involved in certain types of inflammatory diseases by orchestrating the functional phenotype of CD103+ DCs subsets.

## Introduction

Dendritic cells (DCs) is well known as the professional antigen-presenting cells, uptaking and processing pathogenic substance, and presenting the antigen to T cells through peptide–MHC complex (1). In addition to antigens presentation, DCs also play a crucial role in regulating immune response by the co-stimulatory molecules interaction, which provides the second signal in the process of T cell activation and proliferation (2). A number of studies have shown that inhibition or depletion of costimulatory molecules on DCs can improve activity of inflammatory diseases (3–6). Beyond that, there are increasing evidences indicating that DCs also have the capability to induce regulatory T (Treg) cells differentiation, which harness the immune homeostasis (7, 8).

Interleukin (IL)-4, the best-characterized member of the type 2 immune response cytokines, was produced by various types of cells including CD4+ T cells, natural killer T cells, eosinophils, and activated mast cells (9). In the meantime, the function of IL-4 is not yet fully known as the extremely broad distribution of IL-4 receptors (10). Previous studies have shown that IL-4 is the key pro-inflammatory cytokine in the pathological progression of atopic dermatitis, allergic rhinitis, COPD, and cancer (11). Besides, IL-4 has also been implicated in inflammatory bowel diseases (IBDs) (12, 13). Numerous studies have shown that the alteration of T cell polarization could ameliorate the development of disease. It has previously been demonstrated that the development of atherosclerosis and allograft rejection, resulting from Th1 immune response, can be reduced by promoting Th2 differentiation (14). However, the abnormally upregulated Th1 and Th2 immune responses can be observed simultaneously in IBDs (12, 15). Until now, the mechanism in this immune dysfunction remains elusive. Recently, it has been shown that the presence of IL-4 in the initial DCs activation could lead to a dominant Th1 immune response, which had a protective effect in *Leishmania*-infected mice (16). In addition, neutralization of IL-4 abrogated IL-12 secretion in a coculture system of human DCs and Th2 cell (17). Furthermore, high IL-12 and low IL-10 expressions were observed in DCs generated under high concentrations of IL-4 (18). The DCs function altered by IL-4 might explain the mixed Th1/Th2 immune response in IBDs.

CD103+ DCs play a crucial role in alleviating the pathological progression of IBDs through promoting the *de novo* generation of Treg cells by a indoleamine 2,3-dioxygenase (IDO) mechanism (19, 20). IDO is a cytoplasmic rate-limiting enzyme involved in the catabolism and utilization of tryptophan, which converts tryptophan into *N*-formylkynurenine and subsequently kynurenine (21). Recent studies revealed that IDO produced by DCs play a critical role in immune tolerance (22, 23). Our previous study also demonstrated that the protective effect of IL-33 is predominantly dependent on Treg cell expansion, which was closely associated with upregulation of CD103+IDO+ DCs (24). Epithelium cell-derived TGF-β and retinoic acid (RA) were found to be required for CD103+ DC tolerogenic phenotype conversion, and epithelium cell from IBDs patients showed an impaired function in CD103+ DC conversion (20), which result in a diminishment of CD103+ DCs in IBDs (25, 26). Although abnormalities in both Th1 and Th2 immune responses were detected in ulcerative colitis (UC) patients, the Th2 immune response is considered to play a predominant role (15). However, very little is known about the exact role of Th2 immune response in the DCs conditioning during the development of UC. Here, we presented evidence that IL-4 revealed suppressive role on CD103+ DCs conversion. Under high IL-4 circumstance, diminishment of CD103+ DCs was observed in mice bone marrow-derived DCs (BMDCs) and human monocyte-derived DCs (MoDCs), which also showed a weaken potency to simulate Treg cells differentiation. Thus, in addition to impaired intestinal epithelium function in CD103+ DCs conversion, the inflammatory IL-4 also inhibits CD103+ DCs induction, which retard Treg cells differentiation and exacerbate the progress of disease.

## Materials and Methods

### Animals and Human Subjects

Female C57BL/6 mice of 6–8 weeks old were purchased from the Animal Center of Xiamen University (Fujian, China). The mice were housed in the specific pathogen-free facility at the Animal Center of Xiamen University for at least 1 week before inclusion in experiments. All experimental procedures involving mice were approved by the Animal Care and Use Committee of Xiamen University and were carried out in accordance with the recommendations of Animal Care and Use Committee of Xiamen University. Written informed consent was obtained from healthy volunteers (*n* = 10) in accordance with the Declaration of Helsinki. The study protocol was approved by the Ethics Committee, Tongji Medical College, Huazhong University of Science and Technology. The methods were carried out in accordance with the approved guidelines.

### Induction of Colitis

Dextran sulfate sodium (DSS)-induced colitis was induced in C57BL/6 mice as described elsewhere (27). Briefly, the mice were fed DSS (mol wt. ~40,000; Sigma) 2% (wt/vol) dissolved in sterile distilled water for 7 days, followed by a period of 10 days of water without DSS. Mice received four cycles of DSS treatment, and animals were sacrificed. The serum and mesenteric lymph nodes were harvested and analyzed.

### Reagents

All recombinant cytokines in *in vitro* experiments were obtained from Peprotech (London, UK). The antibodies in this study were purchased from commercial companies. Anti-mouse CD4, anti-mouse IFN-γ, anti-mouse CD25, anti-mouse Foxp3, anti-mouse CD11c, anti-mouse CD80, anti-mouse CD86, anti-mouse MHC-II, anti-mouse CD103, anti-human CD4, anti-human IFN-γ, anti-human CD25, anti-human Foxp3, anti-human CD11c, anti-human CD80, anti-human CD86, anti-human CD83, and anti-human CD103 were obtained from ebioscience (CA, USA). Anti-mouse IDO and anti-human IDO were purchased from Biolegend (CA, USA) and R&D (MN, USA), respectively.

### Mouse BMDCs and Human MoDCs Generation

Bone marrow-derived DCs (BMDCs) were generated from the mice bone marrow cells as described previously (28). Briefly, BMDCs were propagated from C57BL/6 mouse at 5 × 10^5^/ml cells in the presence of GM-CSF (10 ng/ml) and various concentration of IL-4 (2, 5, and 10 ng/ml). Half of the supernatant was replaced by same volume of fresh medium containing amount of GM-CSF and IL-4 at days 3 and 5. BMDCs were harvested at day 7 for further study. Peripheral blood samples were collected in anticoagulant tubes from healthy volunteers, and then peripheral blood mononuclear cells (PBMCs) were isolated by standard Ficoll-Hypaque density-gradient centrifugation. CD14+ monocytes were purified from PBMCs using positive selection with CD14+ Cell Isolation Kit human (Miltenyi Biotec, Bergisch Gladbach, Germany). The purity of T cells was >95% as determined using flow cytometry. MoDCs were propagated from CD14+ monocytes under GM-CSF (50 ng/ml) and at various concentration of IL-4 (5, 10, and ng/ml). MoDCs were collected after 6 days of culture.

### T Cells and DCs Coculture

Naïve CD4+ T cells were isolated from splenic cells of normal mice and PBMCs of healthy volunteers by naïve CD4+ T cells negative selection (Miltenyi Biotech, Bergisch Gladbach, Germany). Then the naïve CD4+ T cells were labeled with CFSE and then were cocultured with BMDCs or MoDC. A soluble anti-mouse CD3 or anti-human CD3 (0.5 μg/ml) antibody was added to the T cells/DCs cultural medium. The cells and supernatants were harvested after 5 days and followed by proliferation assay by flow cytometry. The Th1 and Treg cells differentiation were analyzed by intracellular flow cytometry analysis. The 1-MT (25 μM) purchased from Sigma-Aldrich (Shanghai, China) was added to some culture medium for inhibiting the IDO activity. CD103− DCs were isolated from DCs generated under low concentration of IL-4 by negative magnetic beads by the following steps. Biotin-conjugated anti-CD103 antibody was used, then anti-biotin-beads (Miltenyi Biotech, Bergisch Gladbach, Germany) were performed in accordance with manufactory instructions.

### Pinocytosis Assay

The pinocytotic activities of BMDCs and MoDC were measured as described previously (29). Briefly, BMDCs and MoDCs (2 × 105/ml) were incubated with FITC-dextran (1,000 μg/ml) (Sigma-Aldrich, Shanghai, China) for 3 h at 37°C, and then the DCs were washed twice with PBS. Cells were collected and analyzed by flow cytometry on Coulter Beckman. The mean fluorescence intensity of cells incubated with FITC-dextran at 4°C was set as a fluorescence background.

### Flow Cytometry

After propagation, the BMDCs and MoDCs were collected for surface co-stimulatory molecules staining. The IDO staining was performed after DCs treated by Fix/Perm Buffer Set (ebioscience, CA, USA). For cytokines intracellular staining, the cells were obtained from the coculture medium and were stimulated with 20 ng/ml PMA and 1 μg/ml ionomycin (Sigma-Aldrich, Shanghai, China) plus 2 μm of Monesin (Sigma-Aldrich, Shanghai, China) for 4 h following fluorescence-conjugated anti-CD4 antibody staining. IFN-γ and Foxp3 staining was performed in accordance with the manufacturer’s instructions (ebioscience, CA, USA).

### ELISA Assay

Blood samples were collected by cardiac puncture and placed at room temperature for 30 min before centrifugation. The serum was stored at −80°C until analyzed. The levels of IL-4 were determined by ELISA kits (ebioscience) according to the manufacturer’s instructions.

### Western Blots Assay

Total proteins extracted from BMDCs or MoDCs were performed to immunoblot assay conducted as described previously (24). The primary antibody anti-IDO (Abcam, MA, USA) and anti-β-actin (Santa Cruz Biotechnology, Santa Cruz, CA, USA) were used to probe the blots. After incubating with secondary antibody-conjugated horseradish peroxidase, the immunoreactivity was detected by an ECL system (Thermofisher, MA, USA).

### Statistical Analysis

All data were analyzed in GraphPad Prism5. The data are presented as means ± SD. Statistical differences were determined by Student’s *t*-test. Two-sided *p* < 0.05 was considered as significant.

## Results

### High Concentration of IL-4 Promotes a Mature DCs Phenotype from Precursor Cells

**Figure 1:**
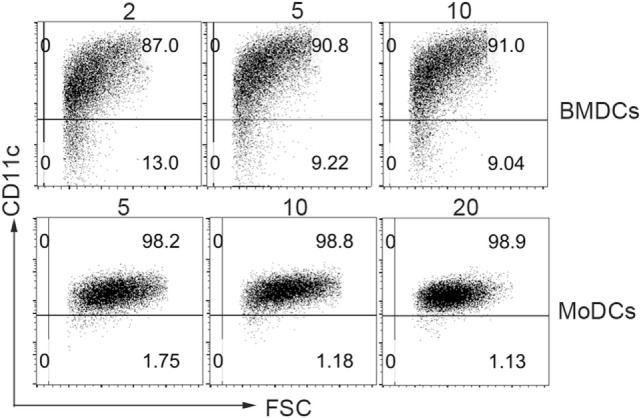
**No difference in CD11c+ dendritic cells (DCs) differentiation from precursor cells by various interleukin (IL)-4 concentrations**. To determine the different dose of IL-4 in bone marrow-derived DCs (BMDCs) and monocyte-derived DCs (MoDCs) differentiation, BMDCs and MoDCs were propagated in various IL-4 concentrations (nanograms per milliliter) as described in Section “Materials and Methods.” After 7 days of BMDCs and 6 days of MoDCs culture, the cells were harvested for CD11c staining, which were analyzed by flow cytometry. The CD11c+ DC were gated for analyzing. Data are representative of three independent experiments.

**Figure 2:**
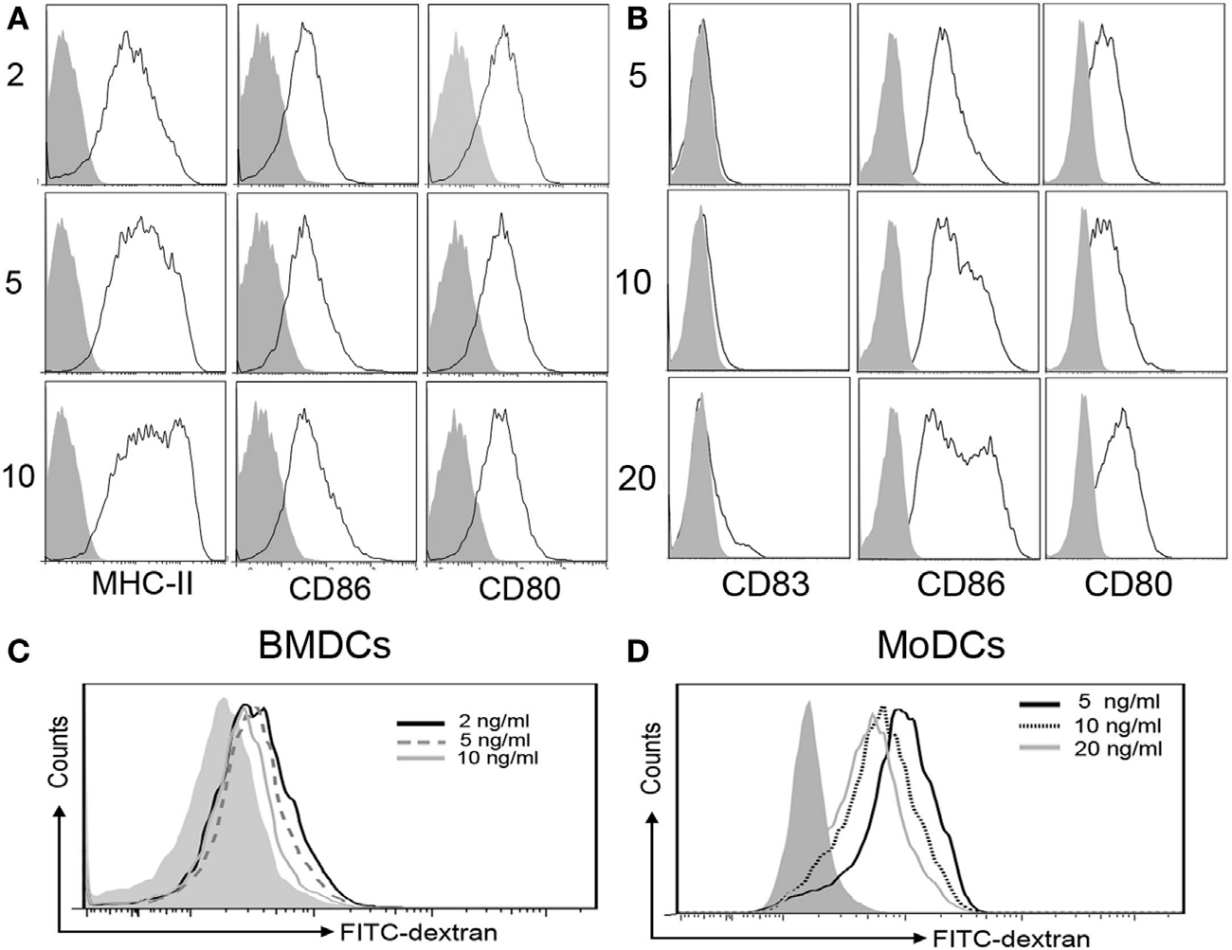
**High interleukin (IL)-4 concentration promotes a mature dendritic cells (DCs) phenotype from precursor cells**. DCs were propagation under various concentration of IL-4 as described in Section “Materials and Methods,” and then the cells were harvested for costimulatory molecules expression analysis by flow cytometry. **(A)** The surface expression of CD80, CD86, and MHC-II on the bone marrow-derived DCs (BMDCs) differentiated from IL-4 (2, 5, and 10 ng/ml, respectively) were plotted in histograms. **(B)** CD80, CD86, and CD83 expressions on monocyte-derived DCs (MoDCs) generated from IL-4 (5, 10, and 20 ng/ml, respectively) were detected. **(C,D)** Pinocytotic activities of BMDCs and MoDCs, which are generated from various IL-4 concentrations as shown above, were represented by mean fluorescence intensity of cells incubated with FITC-dextran. The plots shown were obtained from CD11c+ gate. Data are representative of three independent experiments.

Th2 immune response plays a crucial role in the pathogenesis of many diseases. It is well known that IL-4, the core cytokine in Th2 immunity, is essential for DCs differentiation. To mimic the inflammatory milieu, different concentrations of IL-4 were used in these experiments. There were no significant differences in the yield of BMDCs and MoDCs generation (Figure 1). In comparison to DCs generated under high IL-4 concentrations, DCs generated from low IL-4 concentrations displayed an immature phenotype. Co-stimulatory molecules on BMDCs and MoDCs were markedly higher in high-dose group, when compared with low dose of IL-4 treatment (Figures 2A,B; Table 1). Furthermore, under the condition of low IL-4 concentration, the BMDCs and MoDCs exhibited an increased pinocytotic ability (Figures 2C,D; Table 1), which is a character of immature DCs. These data suggest a crucial role of IL-4 in the development of DCs from precursor cells.

**Table 1:** **Analysis of markers expression of dendritic cells (DCs) generated under various concentration of interleukin (IL)-4**.

	Mean fluorescence intensity (MFI)			

Monocyte-derived DCs (MoDCs) (ng/ml)	CD83	CD86	CD80	FITC-dextran
5	5.97 ± 0.49	135.7 ± 18	41.9 ± 8.3	1,205.3 ± 11.0
10	6.93 ± 0.47^a^	175.0 ± 23.9^a^	38.7 ± 4.6	708.3 ± 17.5^b^
20	9.47 ± 0.75^b^	337.3 ± 53.4^b^	65.6 ± 8.3^a^	583.6 ± 4^b^

**Bone marrow-derived DCs (BMDCs) (ng/ml)**	**MHC-II**	**CD86**	**CD80**	**FITC-dextran**

2	277.3 ± 22.5	44.6 ± 0.7	68.2 ± 4.9	388 ± 14.6
5	375.7 ± 63.5	66.3 ± 1.5^a^	62.6 ± 4.5	348.2 ± 10
10	478.7 ± 17.8^b^	84.9 ± 2.6^b^	70.2 ± 1.0	308 ± 14.8^b^

*The BMDCs and MoDCs were generated under GM-CSF and at different concentrations of IL-4 as indicated, which was detailed in Figure 2. MFI of costimulatory molecules was obtained by flow cytometry analysis. The FITC-dextran by DCs pinocytosis was also analyzed by flow cytometry and present as MFI. Data from three independent experiments are presented as mean ± SD. The low concentration of IL-4 was compared with intermediate and high IL-4 groups*.

*^a^p < 0.05*.

*^b^p < 0.01*.

### IL-4 Promotes T Cells Activation through Regulating IDO Expression in DCs

**Figure 3:**
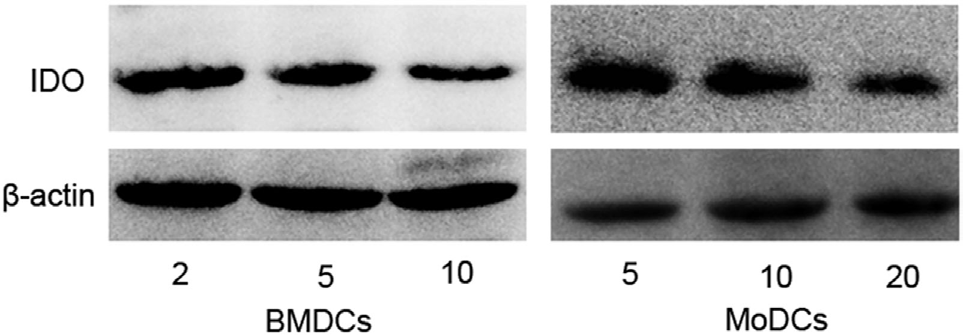
**Interleukin (IL)-4 inhibits indoleamine 2,3-dioxygenase (IDO) expression during dendritic cells differentiation**. Bone marrow-derived DCs (BMDCs) were propagated from bone marrow cells in GM-CSF (10 ng/ml) and different dose of IL-4 (2, 5, and 10 ng/ml) until day 7. Monocyte-derived DCs (MoDCs) were generated from CD14+ monocytes in GM-CSF (50 ng/ml) and various dose of IL-4 (5, 10, and 20 ng/ml) until day 6. After differentiation, total protein from BMDCs and MoDCs were extracted and subjected to assess IDO expression by immunoblot. All data are representative of one of the three independent experiments.

**Figure 4:**
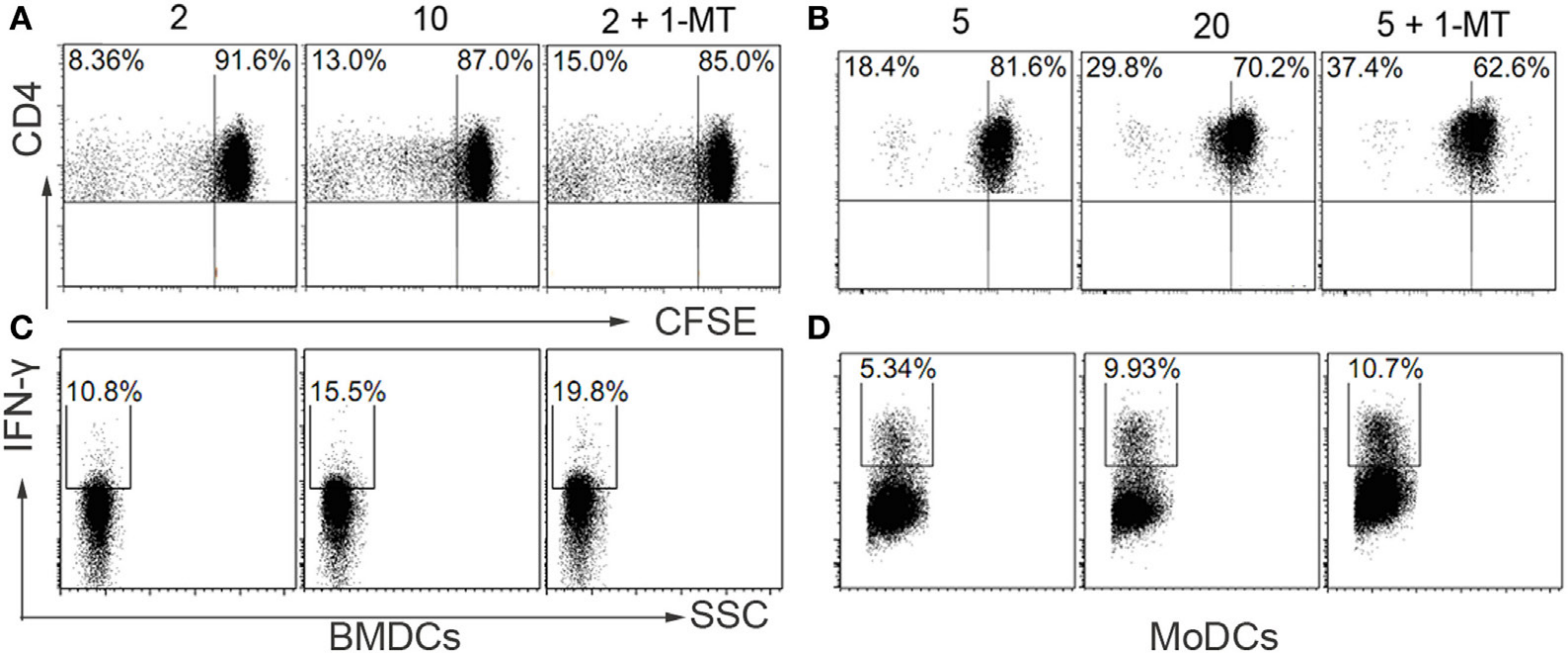
**Interleukin (IL)-4 affects the capability of dendritic cells (DCs) in T cell activation through regulating indoleamine 2,3-dioxygenase (IDO) activity**. Bone marrow-derived DCs (BMDCs) and monocyte-derived DCs (MoDCs) were generated under various concentration of IL-4 (nanograms per milliliter). Then the BMDCs and MoDCs were cocultured with CFSE-labeled naïve CD4+ T cells from normal C57/BL6 mice or healthy volunteers, respectively, with or without the presence of IDO inhibitor (25 μM). After 5 days, the cells were harvested and analyzed by flow cytometry. **(A,B)** The CD4+ T cells proliferation was evaluated by cell division. Results are represented as mean ± SD. **(C,D)** The CD4+ T cells were also collected and analyzed for type 1 cytokines IFN-γ expression by flow cytometry. Results are represented as mean ± SD. The data shown are representative one of the three separate experiments.

Interestingly, previous studies have demonstrated that high concentration of IL-4 augmented IL-12 production and diminished IL-10 expression in DCs, which led to a Th1 immune response (17, 18). IDO secreted by DCs can degrade the amino acid tryptophan, which is essential for T cells proliferation, and exert an inhibitory role in T cells activation (23). However, the effect of IL-4 on IDO expression in DCs was not clearly elucidated. In this study, we observed that IDO expressions in DCs were significantly decreased under the high concentration of IL-4 when compared with low IL-4 (Figure 3). To further explore the role of IDO+ DCs regulated by IL-4 in T cell immune response, the DCs were cocultured with T cells, and the proliferation was investigated. Flow cytometry analysis revealed a high proportion of dividing T cells in DCs differentiated under high-dose IL-4. Conversely, BMDCs and MoDCs differentiated under low-dose IL-4 exerts a gentle action on T cells division (Figures 4A,B). As expected, the neutralization of IDO enzymic activity of low-dose educated DCs by 1-MT led to a markedly enhanced capability in T cells proliferation (Figure 4A). What’s more, when compared with that in high IL-4 group, low-dose IL-4-generated DCs showed a weaker effect on priming naïve T cells to Th1 expansion. This effect was also obviously enhanced by IDO inhibitor 1-MT (Figures 4C,D). These results revealed that IL-4 can regulate IDO expression and suggested that an IL-4-dominated inflammatory cytokine milieu was instructive for immune responses activation through regulating DCs functions.

### IL-4 Retards CD103+ DCs Differentiation

**Figure 5:**
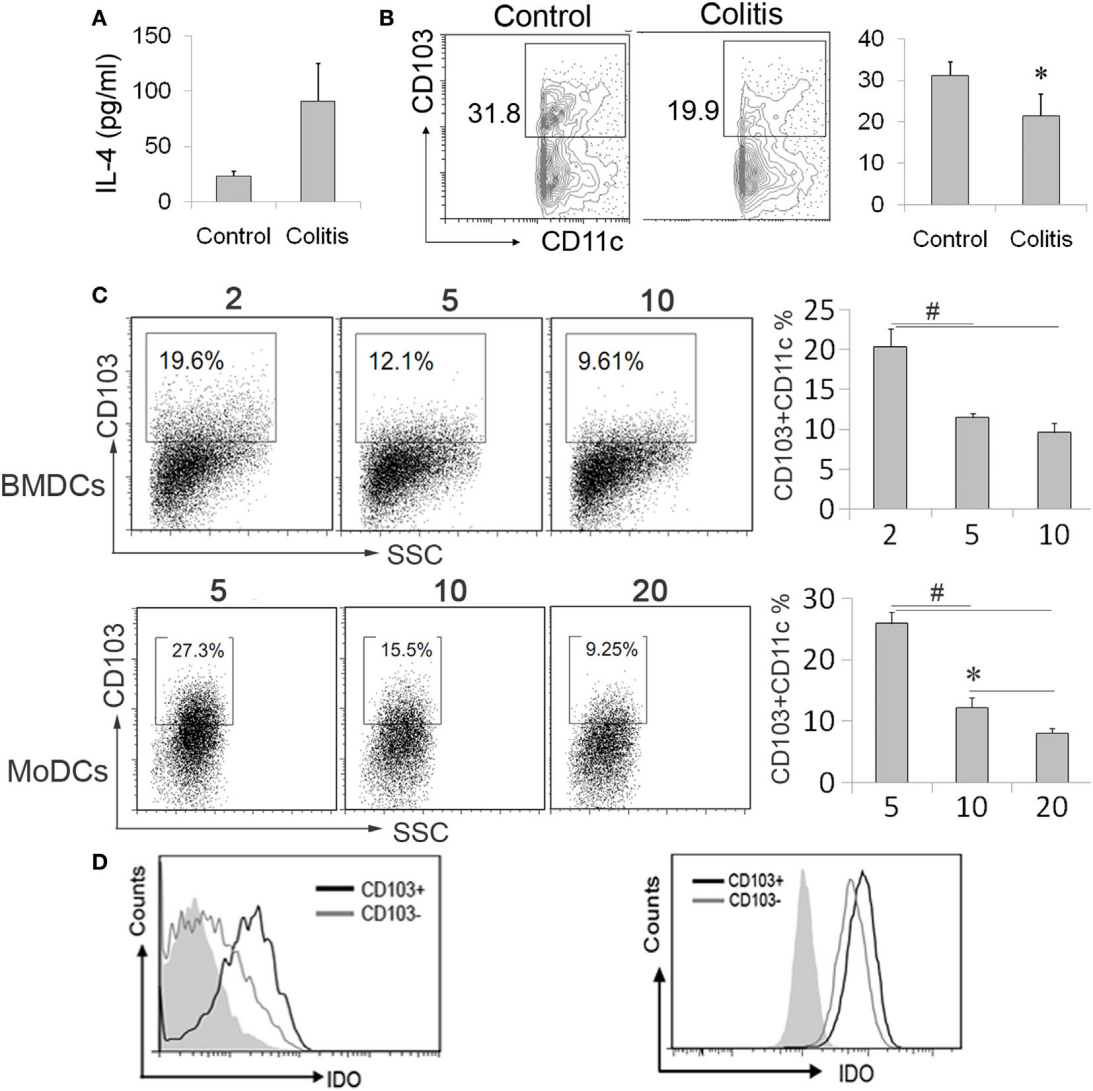
**CD103+IDO+ dendritic cells (DCs) differentiation was affected by interleukin (IL)-4**. **(A,B)** Mesenteric lymph nodes (MLN) cells and serum were collected from the colitis mice after administration of DSS water or normal mice. The interleukin (IL)-4 levels in sera were detected by ELISA assay. **(A)**. MLN cells were analyzed by flow cytometry for CD103+ DCs. The data are presented as the percentage of CD11c+ gate **(B)**. The data represent mean ± SD (n = 4–6/group). **(C,D)** The differentiated bone marrow-derived DCs (BMDCs) and monocyte-derived DCs (MoDCs) from precursor cells in the presence of different IL-4 concentrations (nanograms per milliliter), followed by flow cytometry analysis of CD103 expression **(C)**. The expression of indoleamine 2,3-dioxygenase (IDO) in the subpopulation of BMDCs and MoDCs by CD103 was determined by flow cytometry. The CD11c positive gate was analyzed **(D)**. Data are from one experiment representative of three. Results are represent as mean ± SD. *p < 0.05, ^#^*p* < 0.01.

**Figure 6:**
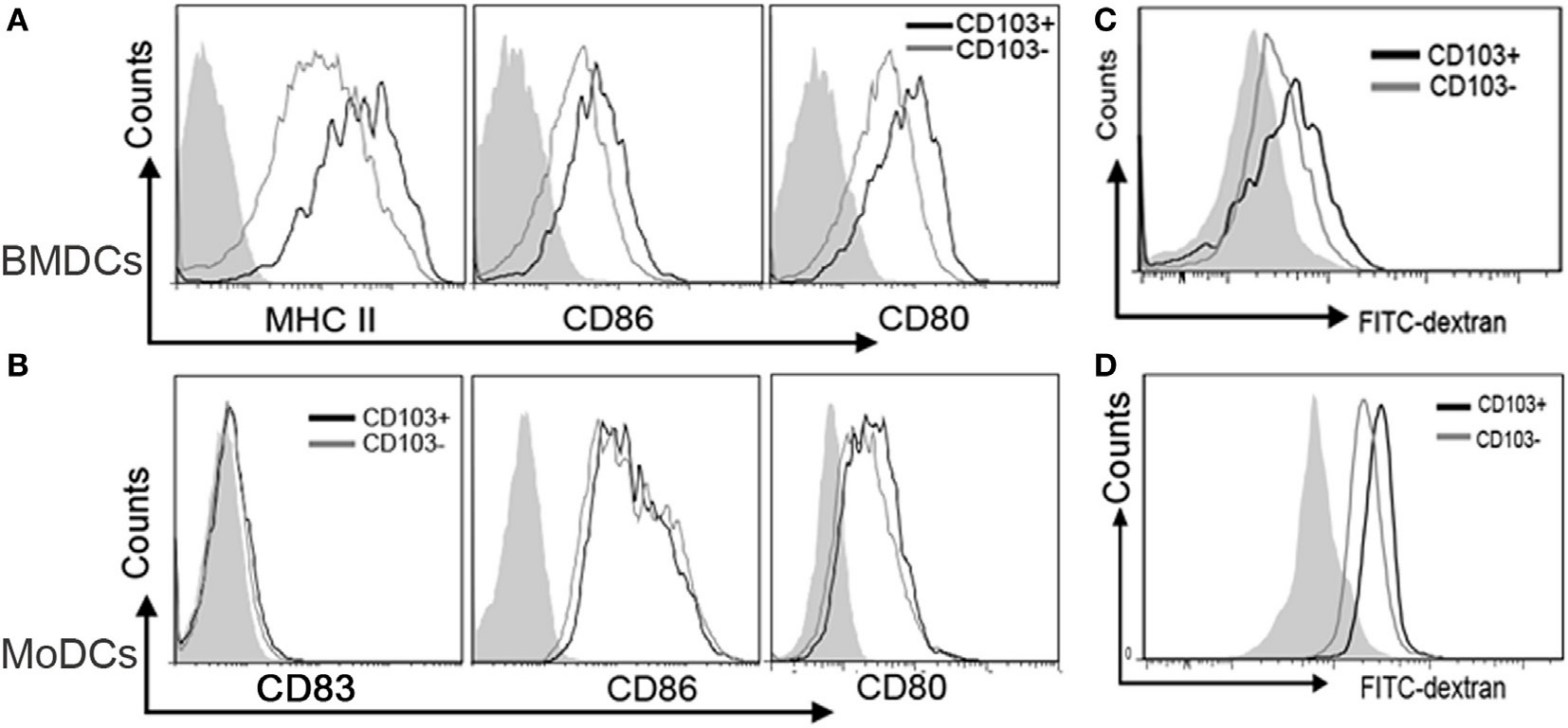
**The phenotype of CD103+ dendritic cells (DCs) differentiated from precursor cells**. DCs were propagation under 2 ng/ml interleukin (IL)-4 for bone marrow-derived DCs (BMDCs) and 5 ng/ml IL-4 for monocyte-derived DCs (MoDCs) in the presence of GM-CSF as described in Section “Materials and Methods,” and then the cells were harvested and analyzed by flow cytometry. **(A)** The surface expressions of CD80, CD86, and MHC-II on the CD103− and CD103+ BMDCs were detected and plotted in histograms. **(B)** CD80, CD86, and CD83 expressions on CD103− and CD103+ MoDCs were analyzed. **(C,D)** Pinocytotic activities of CD103− and CD103+ of BMDCs and MoDCs were represented by mean fluorescence intensity (MFI) of cells incubated with FITC-dextran. Data are representative of three independent experiments.

Studies have shown that CD103+ DCs in the gut showed a high IDO expression (19). In line with increased IL-4 expression in IBDs described by other reports (12, 30), we also observed an increased IL-4 expression in DSS-induced colitis (Figure 5A). Besides, the CD103+ DCs subpopulation was also deceased in colitis mice (Figure 5B). Although above data suggested IDO expression can be regulated by IL-4 and loss of CD103+ DCs during colonic inflammation, the role of elevated IL-4 in the development of gut-associated tolerogenic CD103+ DCs remains unknown. To our expectation, high concentration IL-4 significantly reduced CD103+ DCs proportion in BMDCs and MoDCs (Figure 5C). In parallel with gut CD103+ DCs, the IDO expression was considerably higher in CD103+ DCs when compared with CD103− DCs from BMDCs induced by IL-4 and GM-CSF *ex vivo*. Similarly, a higher expression of IDO was also observed in CD103+ DCs from human MoDCs (Figure 5D). These data indicated that IL-4 involved in the process of CD103+IDO+ DCs education, suggesting that elevated IL-4 expression in IBDs might contribute to pathogenesis of colonic inflammation. However, in contrast to low expression of costimulatory molecules on CD103− DCs, CD103+ DCs highly expressed costimulatory molecules, especially in BMDCs (Figures 6A,B). Although costimulatory molecules commonly facilitated immune response, CD80 and CD86 are also essential for Treg cells development and proliferation in obese mice and humans, which revealed a protective effect on adipose inflammation (7, 8). Notably, CD103+ DCs derived from precursor cells *in vitro* displayed an immature character, as evidenced by a strong pinocytotic ability (Figures 6C,D).

### Impaired Treg Cells Differentiation in DCs Generated in a Low IL-4 Environment due to the Loss of CD103+ DCs

**Figure 7:**
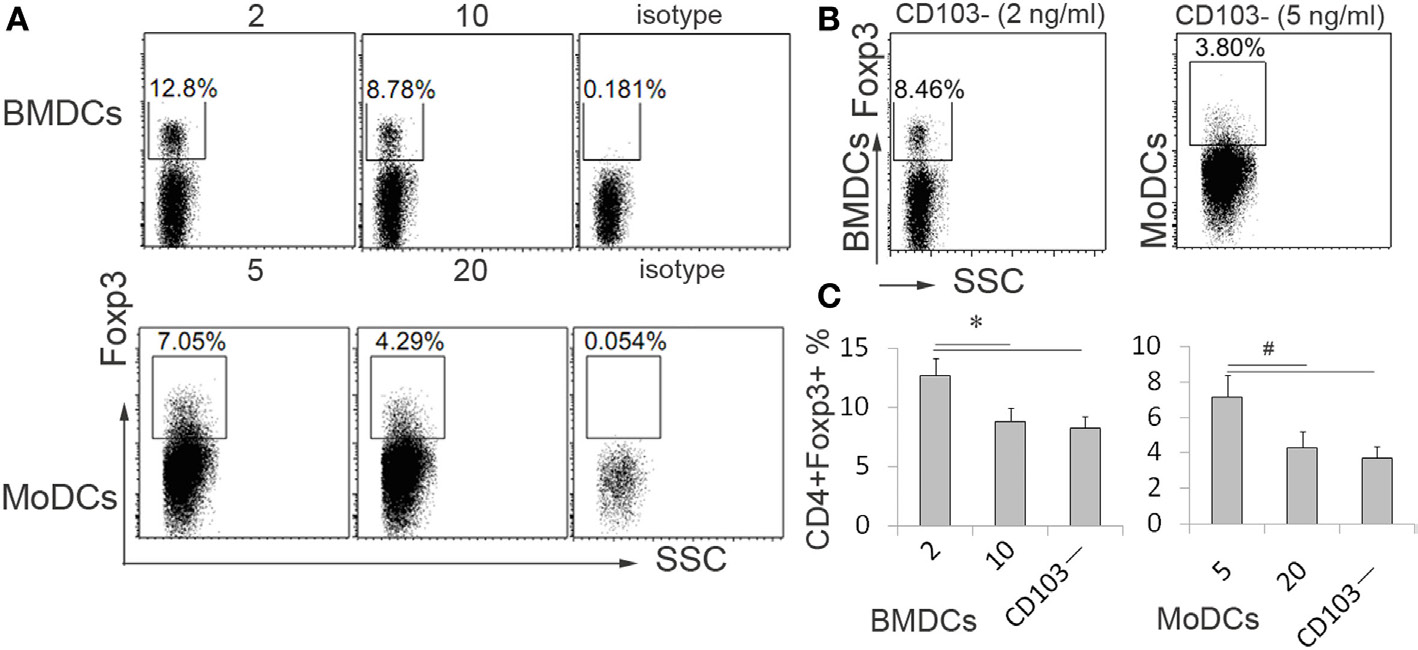
**The potential of dendritic cells (DCs) generated under low concentration of interleukin (IL)-4 in regulatory T cells differentiation depend on CD103+ DCs**. BMDCs and MoDCs were generated under low (BMDC, 2 ng/ml; MoDCs, 5 ng/ml) and high (BMDC, 10 ng/ml; MoDCs, 20 ng/ml) concentration of IL-4 **(A)**. The CD103− DCs were sorted from DCs which generated under low concentration of IL-4 **(B)**. Then these DCs were subjected to cocultured with naïve CD4+ T cells for 5 days in the presence of anti-CD3 (0.5 μg/ml), respectively. Then the cells were collected for analyzing CD4+CD25+Foxp3+ differentiation by flow cytometry. Results are represented as means ± SD **(C)**. The data shown are representative of one of the three separate experiments. *p < 0.05, ^#^*p* < 0.01.

Numerous studies revealed that CD103+ DCs exert a critical role in immune tolerance through promoting Treg cells expansion in gut immunity, and above results showed that IL-4 affected CD103+ DCs induction and Treg cells *in vitro*. However, the Treg cells expanded by DCs differentiated under various concentrations of IL-4 and CD103+ DCs *in vitro* were still elusive. The sorted naïve CD4+ T cells were cocultured with DCs differentiated under low or high IL-4 concentrations and then analyzed by flow cytometry. Both mouse BMDCs and human MoDCs were capable of priming naïve T cells to Treg cells. In contrast to DCs propagated from high-dose IL-4, DCs differentiated at a low concentration of IL-4 exerted a strong preference for expanding Treg cells (Figures 7A,C). Next, to explore whether the low-dose IL-4-educated DCs in Treg cells expansion was contingent on the high propagation of CD103+ DCs, the CD103− DCs generated under low-dose IL-4 were cocultured with T cells. Expectedly, compared with total DCs generated under low dose of IL-4, CD103 DCs exhibited a significant diminishment of the Treg cells differentiation in both humans and mice experiment (Figures 7B,C). These data clearly demonstrate that the concentration of IL-4 present during differentiation of DC precursors is crucial for the CD103+ DCs development in IBDs.

## Discussion

Type 2 immune response exhibits a strong preference in gut immunity by conferring protection against helminthic infection (31). Beyond that, type 2 immune response is also involved in the process of wound healing in the gut (32, 33). Nevertheless, dysregulated and overreactive Th2 immunity may lead to detrimental inflammation. Th2-induced fibrogenesis can lead to a deleterious consequence by augmenting mucosal and transmural fibrotic process, a prototypic manifestation of IBDs (15, 33). It is well known that IL-4, the core signature of Th2 responses, is highly expressed in certain types of inflammation and also exerts pleiotropic functions due to extremely broad distribution of IL-4 receptors (10). Enormous reports have also provided evidences that IL-4 participates in the pathogenesis of IBDs (34, 35). Oxazolone-induced colitis, resembling the human UC, shows a bias of type 2 immune response. It is noteworthy that oxazolone-induced colitis in mice lacking IL-4Ra or STAT6 was significantly improved (36, 37). Consistently, blockade of IL-4 cytokine activity by IL-4 antibody administration exhibited a protective effect in oxazolone-induced colitis (38, 39). In accordance with previous studies (12, 15), our data also showed that there was an increased IL-4 level in DSS-induced colitis (data not shown). Furthermore, the importance of IL-4 in DSS-induced colitis model was confirmed by using IL-4 knockout mice (40). However, the mechanism of IL-4 in colitis remains to be defined. Robust data have indicated a crucial role for DCs in the pathogenesis (41). In this study, we demonstrate a novel function of IL-4 in regulating the CD103+ DCs differentiation. CD103+ DCs, a critical DCs subset in the gut immune homeostasis and the primary source of IDO, play a preponderant role in IBDs through promoting Treg cells differentiation (42). Our previous study also demonstrated that the administration of rIL-33 favors Treg cells function through upregulation of CD103+ DCs in animal experimental colitis (24). A tendency of decreased number of Treg cells was observed here (data not shown). The number of Treg cells was not significantly changed, which might be due to the Treg cells differentiation affected by many factors *in vivo*. However, their suppressor activity may be abrogated *in vivo* or they are unable to counterbalance the chronic mucosal inflammation in UC (43).

Enormous progress has pinpointed that CD103+ DCs are an crucial character in the development of IBDs through promoting Treg cells differentiation. Furthermore, epithelium cells gained attention owing to its role in secretion of TGF-β and RA, which are required for CD103+ DCs tolerogenic phenotype conversion (20). Indeed, rIL-33 administration resulted in an increased ALDH1A1 and TSLP expression in intestine epithelium cells, resulting in an increased conversion of CD103+ DCs (24). Moreover, in accordance with other reports (25, 26), we also observed loss of CD103+ DCs during colonic inflammation in DSS-induced colitis (data not shown). Notably, epithelium cell from IBDs patients, which were damaged during the abnormal immune response, showed an impaired function in CD103+ DCs propagation (20). Due to the defect in Treg cell differentiation, an impaired conversion of CD103+ DCs in epithelial injury might be an important mechanism for the progression of colitis. In spite of the critical role of CD103+ DCs in the gut immune homeostasis, the understanding of CD103+ DCs conditioning during the process of abnormal immune response in IBDs is yet little to known. Although the beneficial effects of epithelium cells on CD103+ DCs conversion was impaired in the development of IBDs (20), a question remained to be addressed is that whether the epithelium injury is the cause or a consequence of CD103+ DCs loss.

Heretofore, enormous progress has been made in understanding the role of IL-4 in the pathogenesis of IBDs, and the precise mechanism of this cytokine in IBDs is still unclear and needs to be further studied (33). Until now, many cytokines exhibit a strong preference for educating DCs. IL-10-treated DCs followed by LPS stimulation suppressed alloreactive T cells during allograft rejection (44). Furthermore, IL-17 neutralization led to alteration of phenotype and function of DCs and diminishment of Th1 type immune response in the mouse chlamydial lung infection and allograft transplantation (45, 46). Not only that, in a human DCs and Th2 cell coculture system, inhibition of IL-4 activity abrogated IL-12 production (17). Moreover, DCs generated under high concentrations of IL-4 produced high amounts of IL-12 and low IL-10 DCs (18). Although IL-4 acts a crucial role in the pathogenesis of IBDs (33), very little information about its action in DCs education is available. Here, our study presented the evidence that IL-4 exerts a regulatory role in CD103+ DCs differentiation. Moreover, there was a dose-dependent effect for IL-4 in the inhibition of CD103+ DCs differentiation. These data might provide explanation why CD103+ DCs were decreased in DSS-induced colitis, where IL-4 was upregulated. Furthermore, following the loss of CD103+ DCs, Treg cell differentiation was causally defective. Our study also provided an evidence that the suppressive effect of low-concentration IL-4-generated DCs primarily depends on IDO, the key regulator of gut CD103+ DCs (19). To our surprise, the costimulatory molecules on CD103+ DCs were significantly increased when compared with CD103− DC. The costimulatory molecules play critical role in initiating the immune response (2), and B7/CD28 interaction provides the important second signal for T cell activation (47–49). Nevertheless, CD80/CD86 is also essential for Treg cells development and proliferation in obese mice and humans and inhibits adipose macrophage inflammation (7). Thus, the concrete function of upregulated costimulatory molecules on CD103+ DCs needs to be further explored.

In conclusion, we provided novel insights into the loss of CD103+ DCs in IBDs, which might be a result of elevated IL-4 expression. Beyond that, our study supported a new relationship among IL-4, CD103+ DCs, and epithelial cells in the pathogenesis of IBDs: in the initial phase of colitis, abnormal Th2 type immune response causes an increased IL-4 expression that leads to loss of tolerogenic CD103+ DC and Treg cells dysfunction. Furthermore, the injury of epithelium resulted from abnormal immune response, in turn, worsens the abnormal immune response due to impaired capability to induce CD103+ DCs conversion. Therefore, IL-4 plays a crucial in the development of colitis.

## Author Contributions

LD conceived the project, designed and carried out some experiments, analyzed all data, and wrote the paper; JC were responsible for design and performance of experiments and analyzed data and wrote the paper; LT performed all the experiments, analyzed all data, generated figures of the data, and performed statistical analysis; HZ performed all the experiments, analyzed all data, and generated figures of the data. All the authors read, critically revised, and agreed to be accountable for the content of manuscript.

## Conflict of Interest Statement

The authors declare that the research was conducted in the absence of any commercial or financial relationships that could be construed as a potential conflict of interest.
